# Research on Segmentation Method of Maize Seedling Plant Instances Based on UAV Multispectral Remote Sensing Images

**DOI:** 10.3390/plants13131842

**Published:** 2024-07-04

**Authors:** Tingting Geng, Haiyang Yu, Xinru Yuan, Ruopu Ma, Pengao Li

**Affiliations:** 1 School of Surveying and Land Information Engineering, Henan Polytechnic University, Jiaozuo 454000, China; 212204020017@home.hpu.edu.cn (T.G.); 212204020077@home.hpu.edu.cn (X.Y.); 212204020039@home.hpu.edu.cn (R.M.); 212104020002@home.hpu.edu.cn (P.L.); 2 Key Laboratory of Mine Spatio-Temporal Information and Ecological Restoration, Ministry of Natural Resources, Henan Polytechnic University, Jiaozuo 454000, China

**Keywords:** multispectral data, maize seedlings, instance segmentation, YOLOv8 model

## Abstract

The accurate instance segmentation of individual crop plants is crucial for achieving a high-throughput phenotypic analysis of seedlings and smart field management in agriculture. Current crop monitoring techniques employing remote sensing predominantly focus on population analysis, thereby lacking precise estimations for individual plants. This study concentrates on maize, a critical staple crop, and leverages multispectral remote sensing data sourced from unmanned aerial vehicles (UAVs). A large-scale SAM image segmentation model is employed to efficiently annotate maize plant instances, thereby constructing a dataset for maize seedling instance segmentation. The study evaluates the experimental accuracy of six instance segmentation algorithms: Mask R-CNN, Cascade Mask R-CNN, PointRend, YOLOv5, Mask Scoring R-CNN, and YOLOv8, employing various combinations of multispectral bands for a comparative analysis. The experimental findings indicate that the YOLOv8 model exhibits exceptional segmentation accuracy, notably in the NRG band, with bbox_mAP50 and segm_mAP50 accuracies reaching 95.2% and 94%, respectively, surpassing other models. Furthermore, YOLOv8 demonstrates robust performance in generalization experiments, indicating its adaptability across diverse environments and conditions. Additionally, this study simulates and analyzes the impact of different resolutions on the model’s segmentation accuracy. The findings reveal that the YOLOv8 model sustains high segmentation accuracy even at reduced resolutions (1.333 cm/px), meeting the phenotypic analysis and field management criteria.

## 1. Introduction

Maize, a traditional staple crop [1], is characterized by its diverse attributes, serving as an essential source of both feed and industrial raw materials. It is widely distributed worldwide [2]. Monitoring its growth process is crucial in agricultural production to increase yield and optimize cultivation practices.

However, traditional agricultural monitoring methods are constrained by labor and time costs, making it difficult to efficiently monitor large agricultural fields [3,4]. With the rapid advancements in imaging technology and artificial intelligence, deep learning techniques employing visible light images have been applied to the detection of crop seedlings [5]. The University of Alaska, USA, developed an advanced benchtop phenotyping platform [6] enabling the automated high-throughput testing of stationary plants with precise control over temperature, humidity, and light intensity. The DroughtSpotter [7], produced by PhenoSpex in the Netherlands, and the WPScan [8], produced by WE PROVE SOLUTIONS (WPS), a phenotypic information collection platform, can extract parameters of plant transpiration dynamics and growth status at various stages through image collection. Compared with the above platforms, unmanned aerial vehicles (UAVs), which are not affected by ground conditions and can efficiently collect high-resolution images, have become a popular choice for crop detection research in recent years [9,10,11,12,13]. In agricultural monitoring, UAV-mounted multispectral sensors yield more accurate information in the infrared band compared to visible imaging, offering farmers and agronomists valuable insights into plant conditions. Relative to hyperspectral sensors, multispectral sensors are more cost-effective and lighter, facilitating their integration into UAVs with smaller payloads and broadening the application range of various UAV types in agriculture [14,15,16]. Zhao [17] et al. used UAV multispectral imagery to make a diagnosis of nitrogen nutrition index (NNI) in cotton. Hu [18] explored the potential of UAV technology in rapidly producing maps of leaf chlorophyll content (LCC) and fractional vegetation cover (FVC) from maize canopy orthophotos to evaluate maize maturity. Han [19] identified a strong correlation between the maize height estimated from UAV imagery and the actual ground truth height. While existing studies have analyzed maize biomass, chlorophyll content, and height, research on the accurate segmentation and leaf area analysis of individual maize seedlings remains scant.

With the rapid advancement of computer vision and deep learning technologies, image-based plant instance segmentation has emerged as a critical research tool, offering enhanced accuracy for agricultural production [20]. Turgut [21] used deep learning architecture based on attention mechanism to achieve plant organ segmentation by extracting contextual features and performing feature propagation to process point regions in a hierarchical manner. Lu [22] used UAV aerial images to obtain information such as canopy area, canopy width, location, etc., and proposed an unsupervised image segmentation method for the fast acquisition of fruit tree canopies under natural lighting conditions. Ren [23] first proposed a recurrent neural network-based recurrent instance segmentation algorithm to achieve leaf segmentation, but the final segmentation effect was very unsatisfactory; then, it was improved by using Conditional Random Fields (CRFs) [24] as the post-processing step of the network: the leaf segmentation effect was improved, and the evaluation metrics symmetric best dice (SBD) [25] was improved by about 10%. Yin [26] proposed the use of a watershed algorithm to complete the segmentation of individual leaves in an image, which in turn enables leaf counting. Zhang et al. [27] employed an unmanned aerial system to gather cabbage germplasm resources, achieving individual cabbage segmentation and enabling the estimation of its width, length, and relative chlorophyll content. Based on a comprehensive review of the existing literature, there is currently a lack of specialized research on the instance segmentation of early-stage maize seedlings. In addition, existing algorithms are not fully applicable to early-stage maize detection tasks.

The application of deep learning techniques necessitates a substantial volume of trainable data to fully leverage the sophisticated feature extraction and image analysis capabilities of deep convolutional neural networks [28,29]. Training samples for image instance segmentation necessitate pixel-level mask information, consequently escalating the cost of manual annotation. This limitation hampers the accurate representation of the plant’s phenotypic information. Qiang [30] achieved the segmentation of green leafy vegetable instances using deep convolutional neural networks; however, the model’s training demands extensive pixel-level mask-labeled data, with labeling often requiring several hours per image, thereby impeding the practical application of these algorithms. Bearman [31] et al. analyzed sample labeling methods, finding pixel-level annotation of an image, on average containing 2.8 objects, requires approximately 4 min, with the time cost for annotating single plant trait features being two to three times higher. To mitigate the manual annotation costs, Zhao introduced a weak label generation method using bounding boxes [32], facilitating the high-precision segmentation of maize seedling images at a reduced annotation time of approximately 2.5 min/sheet. While these methods offer effective solutions for image segmentation, their efficacy in processing field-specific images of young maize seedlings falls short of efficiency requirements. Furthermore, the volume of collected data samples is insufficient to comprehensively represent the diverse scenarios and conditions encountered in field environments.

The YOLOv8 algorithm introduces new features and enhancements that build on the success of previous You Only Look Once (YOLO) versions, with faster detection speed and higher accuracy, providing more advanced technical support for image processing. In contemporary research, the applications of YOLOv8 extend across diverse domains, such as automated waste sorting and detection [33], gravel soil mixture homogeneity assessment [34], pest field management [35], and the real-time detection of high-speed railway components [36], among others. However, despite YOLOv8’s impressive achievements in numerous fields, there is a notable absence of detailed research on applying the YOLOv8 model to young maize.

To date, significant advancements have been made in crop instance segmentation using UAV multispectral technology; however, the research in this field continues to confront numerous challenges. (1) The first is the lack of representative example segmentation datasets. Training samples for image instance segmentation necessitate pixel-level mask information, substantially increasing the cost of manual annotation, particularly in plant phenotyping. The diversity and complexity of images, alongside the variability of plant morphology and large sample sizes, make acquiring high-quality, representative datasets exceedingly challenging. (2) Another challenge is the limited applicability of instance segmentation algorithms. Not all existing algorithms are suitable for early-stage maize detection. Owing to the small size of early-stage maize plants and differences in leaf morphology and texture relative to mature plants, some algorithms demonstrate reduced accuracy and robustness in these tasks. Furthermore, most of the existing instance segmentation algorithms are optimized for outdoor scenes and mature crops, and they are ineffective in recognizing and segmenting the small scale, variable growth state and complex environmental disturbances of juvenile maize in farmland, which makes it difficult to meet the demand for the intelligent management of large-area maize farmland and achieve fine management.

In response to the aforementioned challenges, this paper presents the following principal contributions. (1) The first is the construction of an early-stage maize multispectral instance segmentation dataset. Utilizing the Segment Anything Model (SAM) [37] enabled the semi-automatic and efficient annotation of early-stage maize seedlings, thus creating a representative and high-quality dataset for maize seedling instance segmentation. (2) The second is evaluation of the performance of multiple mainstream instance segmentation algorithms on the constructed dataset. In addition, a special performance analysis was conducted for the overdense areas in corn planting to help farmers make more scientific field management decisions, optimize crop growth conditions and ultimately improve corn yield and quality.

## 2. Materials and Methods

The eastern region of Nanzhang Village, Shanyang District, Jiaozuo City, Henan Province, was selected as the research area (35°9′ N, 113°14′ E). The area is characterized by flat terrain, fertile soil, abundant water resources, and favorable agricultural production conditions. As a key contributor to China’s grain production, Henan Province has demonstrated its distinctive characteristics and representation in the field of agricultural development. The research area is primarily devoted to the cultivation of corn, wheat, rice, and other crops. Maize, in particular, was planted on 3864.37 thousand hectares in Henan Province in 2023, underscoring its pivotal role in the local agricultural economy. Nanzhang Village, situated in Shanyang District, Jiaozuo City, is located within the Huang-Huai-Hai Plain, which is classified as belonging to the temperate monsoon climate zone. This climatic zone is characterized by four distinct seasons, sufficient light and abundant heat, which provides an ideal environment for the growth of young maize.

**Figure 1:**
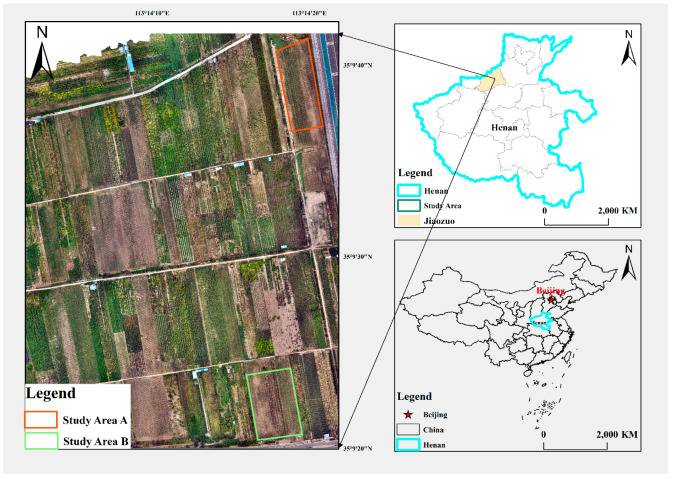
Overview of the study area.

In this study, model training and optimization were conducted in research area A, while experiments on model generalization application were carried out in research area B. The distribution of Study Area A and Study Area B is shown in Figure 1, where the area of Study Area A is 6545 square meters, the maize varieties planted are for Xingmin 176 with self-retaining loose seed, and the maize sowing time is 8 June 2023. Xingmin 176 is a variety that has demonstrated positive performance in Henan Province and has high yield potential based on historical data. Its characteristics include purple leaf sheaths at the seedling stage, green leaf blades, vigorous growth, and a more compact plant shape. Establishing a training and validation dataset based on these characteristics helps to provide more reliable results for practical applications. Research area B covers an area of 5445 square meters and is planted with the Fengshou 2 maize variety, which was sown on 6 June 2023. Selecting different maize varieties for this study enables a deeper understanding of the influence of segmentation algorithm accuracy across various instances, thereby facilitating the optimization of algorithm parameters and model design.

### 2.1. Data Acquisition

The dataset for this study was collected on 4 July 2023, in the eastern cornfield of Nanzhang Village, Shanyang District, Jiaozuo City, Henan Province. Data were collected using a DJI Phantom 4 Multispectral ( DJI-Innovations Inc., Shenzhen, China), with the flight altitude set at 8.5 m, a side-by-side overlap of 65%, and a heading overlap of 65%. The weather on that day was clear and calm, thereby eliminating the possibility of image distortion due to weather conditions, and ensuring an image resolution of 0.4 cm. A total of 2238 aerial photographs were obtained.

### 2.2. Data Preprocessing

**Figure 2:**
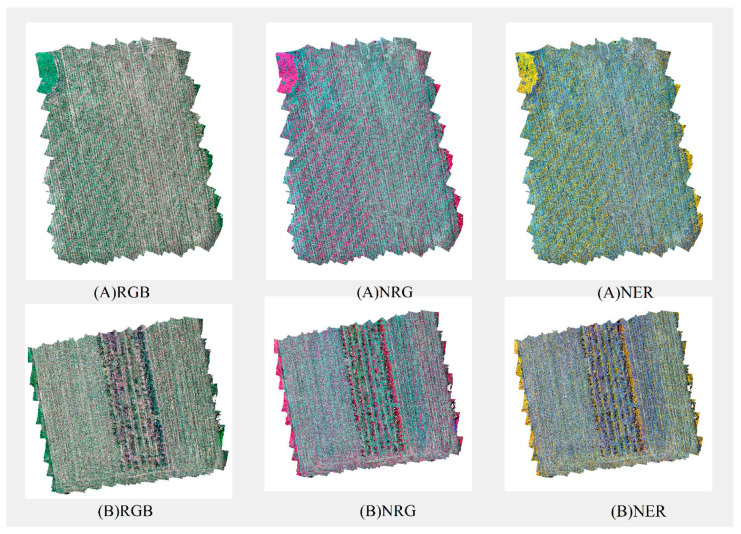
Orthorectified images of Study Area **A** and Study Area **B**.

After ensuring that the image quality of the UAV meets the research requirements, this study used version 2.1 Sablefish of the open source software OpenMVG [38] for Structure from Motion (SfM) processing to reconstruct the multispectral ortho image. By analyzing images from different viewing angles, SfM technology can reconstruct the three-dimensional structure of the scene without additional measurement equipment. OpenMVG is used to extract feature points from the images and calculate the camera pose to generate sparse point cloud data. Based on these data, we construct a Digital Elevation Model (DEM) that describes the elevation information of the surface. Combined with camera pose and automatic calibration parameters, we further compute the orthophoto image of the multispectral band. Finally, orthophoto images of RGB (Red–Green–Blue), NRG (Near Infrared–Red–Green), NER (Near Infrared–Red Edge–Red) and other bands are synthesized, and the results are shown in Figure 2:

### 2.3. Dataset Construction

**Figure 3:**
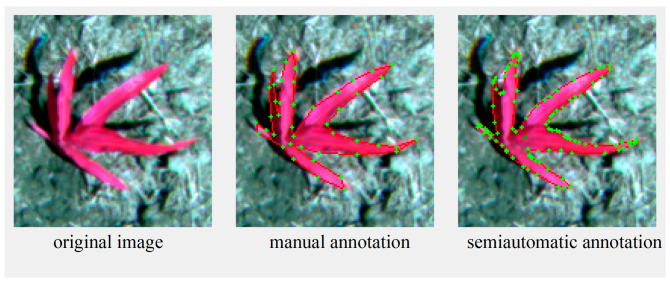
Comparison between traditional manual annotation and semi-automatic annotation based on SAM model. The green plus sign (‘+’) indicates a key turning point in the annotation process.

**Figure 4:**
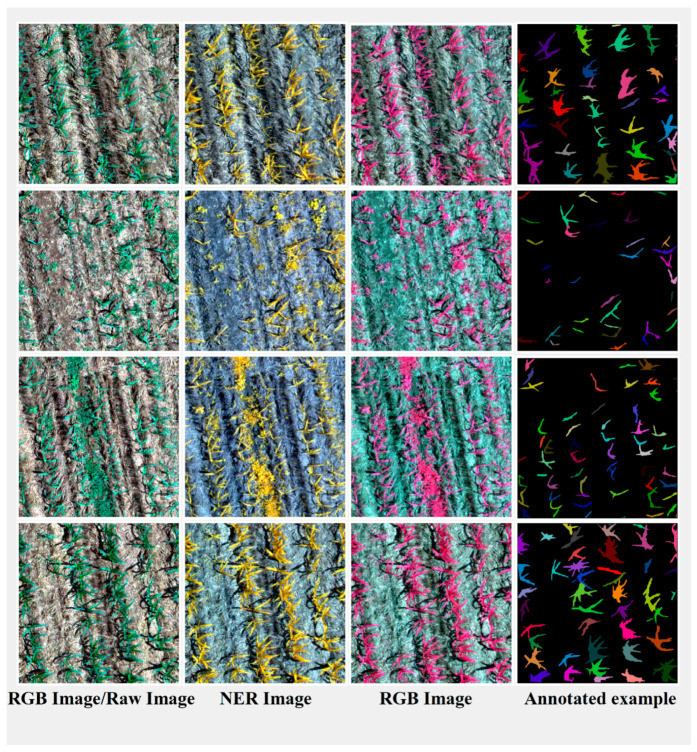
Labeling effect.

In order to ensure the effectiveness of detection and segmentation of small targets, we segmented the orthophoto into 640 × 640-pixel image slices. To avoid missed detections caused by targets located between the slices, we set the overlap rate of the slices to 10%. We utilized the SAM [37] for the semi-automatic labeling of individual maize plants. Compared with the weakly supervised learning method [32], this approach achieves a more accurate delineation of monocot maize through manual intervention. Compared to the traditional manual labeling method, the efficiency of semi-automatic labeling using the SAM-based approach has significantly improved, reducing the average labeling time per maize plant to less than 6 s. As illustrated in Table 1, the reduction in annotation costs further demonstrates the advantages of SAM-based semi-automatic annotation in reducing both time and labor inputs for dataset annotation. As depicted in Figure 3, we compare the traditional manual annotation method with the SAM-based semi-automatic annotation method, where the green plus sign (‘+’) on the red contour line indicates the key turning points in the annotation process. The results indicate that the masks generated by the SAM-based semi-automatic labeling method have finer edges and exhibit higher accuracy compared to those produced by the traditional manual labeling method. The labeling results of some images are shown in Figure 4, where 488 images (80%) from Study Area A were used as the training sample set, and 123 images (20%) were used as the validation sample set. In addition, an independent test set consisted of 723 images from Study Area B. This test set was only used to test the generalization ability of the model and did not participate in the training process of the model. According to the statistical results, a total of 24,064 maize plants were labeled in Study Area A, and 29,226 maize plants were labeled in Study Area B. In order to quantify the number of instances in the training, validation, and testing datasets, we used the COCO dataset’s size classification criterion for targets. According to this criterion, small targets refer to those instances smaller than 32 × 32 pixels, medium targets represent instances between 32 × 32 and 96 × 96 pixels, and large targets refer to those instances larger than 96 × 96 pixels. The specific statistics of the number of instances are shown in Table 2.

### 2.4. The SAM Model

The Segment Anything Model (SAM) [37] is a segmentation model released by Meta in April 2023. It provides the latest results in zero-sample segmentation and is trained on a large-scale SA-1B dataset containing more than 11 million images and 1 billion markers. SAM is capable of segmenting any image or object in any video, eliminating the need for additional training information. The algorithm is utilized not only for satellite image segmentation [39] but also for diverse image segmentation tasks, including medical image segmentation [40], livestock tracking mask extraction [41], and autonomous robot frameworks [42].

**Figure 5:**
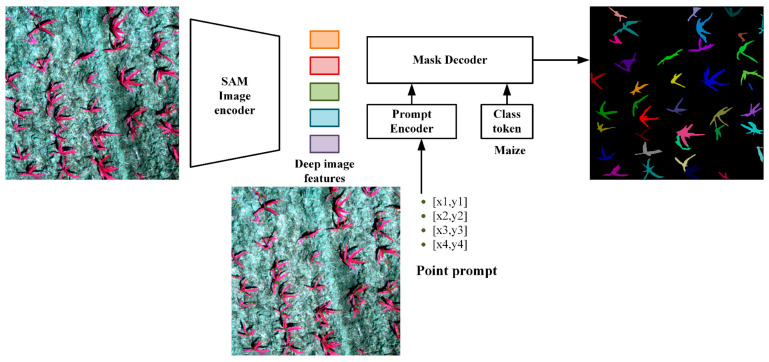
SAM model drawing labeling flowchart.

In this study, the SAM model is employed to perform the rapid, semi-automatic labeling of point cues for young maize in the image, and the labeling process is depicted in Figure 5. The model consists of three components: an image encoder, a prompt encoder, and a mask decoder.

#### 2.4.1. Image Encoder

The image encoder is structured as a Vision Transformer (ViT) [43]-based masked autoencoder (MAE) [44] for extracting image masks from high-resolution image inputs, which are fed into a mask decoder along with cued embeddings that have been feature extracted.

#### 2.4.2. Prompt Encoder

The input prompts of SAM are categorized into two types: one for sparse prompts, such as dots, bounding boxes, and text; and another for dense prompts, such as masks. The sparse cues are processed by the cue encoder to generate cue embeddings; point cues and bounding box cues are transformed through positional encoding, whereas text cues undergo processing by the text encoder in Contrastive Language-Image Pre-Training (CLIP) [45]. Mask cues undergo convolution and are subsequently summed element by element with the image features. In this study, we utilize point cues to activate the SAM’s zero-sample capability. Given a point cue, SAM can automatically segment a corresponding object from an image within seconds. If the mask lacks precision, it can be refined by adding cues to either missing or excessive regions. This mask can be readily converted into an annotation. This process can be regarded as a semi-automatic labeling method for dataset annotation.

#### 2.4.3. Mask Decoder

The mask decoder efficiently converts image features, cue embeddings, and class tokens into masks. This is achieved using the SAM mask decoder, which utilizes a lightweight Transformer [46] decoder block to convert class tokens (young maize seedlings), cue embeddings, and image features into a dynamic classifier. Subsequently, it computes the probability of each pixel in the image to produce a mask.

### 2.5. The YOLOv8 Model

**Figure 6:**
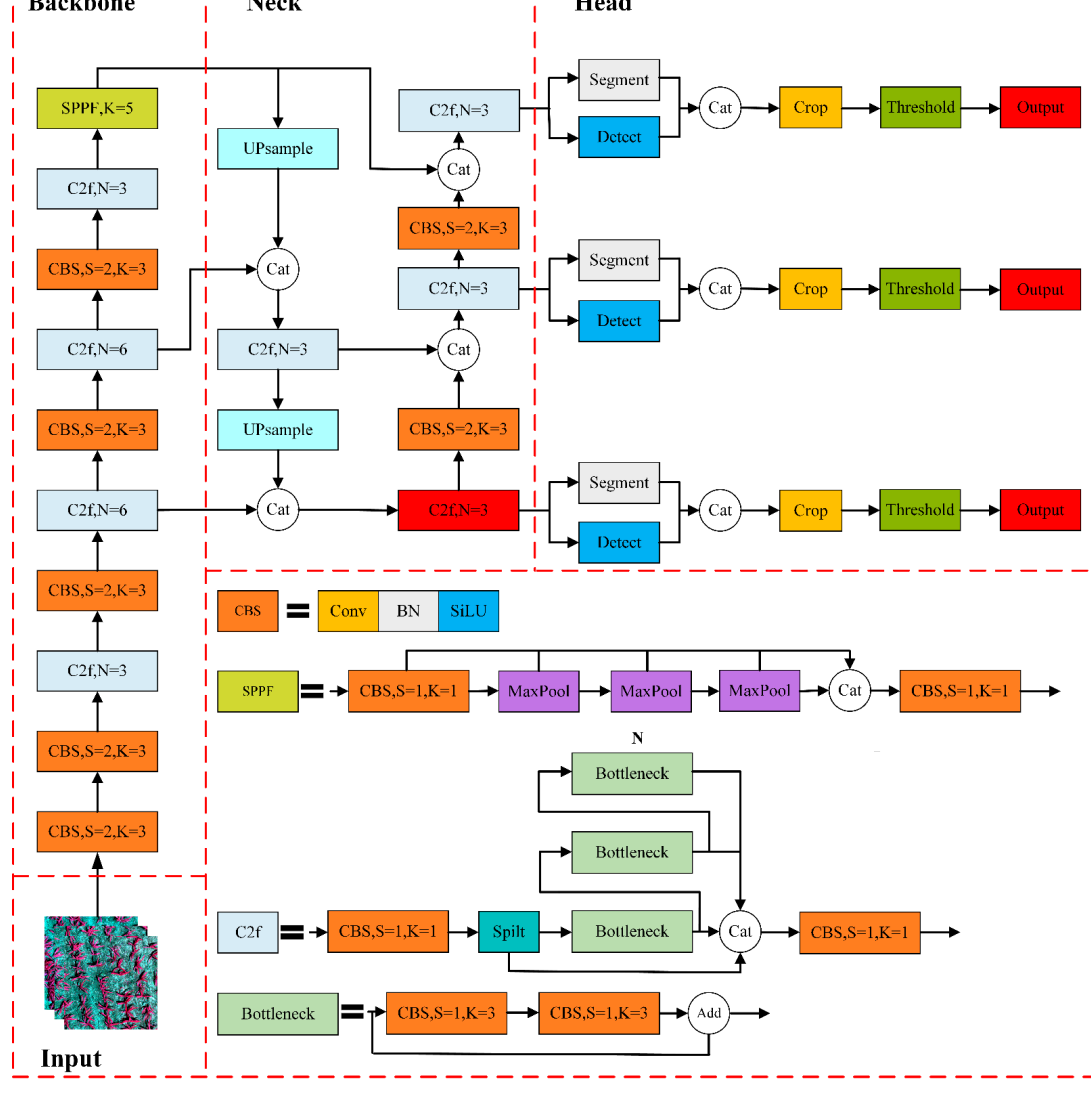
YOLOv8-Seg network structure.

Compared to its predecessors, the YOLOv8 model boasts enhanced speed and accuracy. YOLOv8 is categorized into five distinct model sizes based on network depth and feature map dimensions: YOLOv8n, YOLOv8s, YOLOv8m, YOLOv8l, and YOLOv8x. YOLOv8n (YOLOv8 Nano) is the fastest and smallest, while YOLOv8x (YOLOv8 Extra Large) is the most accurate but slowest among them. According to the network architecture diagram, YOLOv8 can be divided into four parts: Input, Backbone, Neck, and Head. The network architecture of YOLOv8-Seg is illustrated in Figure 6:

#### 2.5.1. Input

The primary objective is to resize images to the required training size and perform data augmentation operations. YOLOv8 utilizes an adaptive image scaling method to resize images adaptively, minimizing the width of black borders to reduce computational overhead in image processing. Data augmentation is a technique for generating additional equally effective data based on a limited dataset without altering the essential information in the images. This approach enhances the model’s generalization ability by compelling it to learn more resilient features. Common data augmentation techniques comprise horizontal/vertical flipping, random cropping, scaling, and rotation, among others. During the training of this study, the Mosaic [47] online data augmentation method was disabled in the final 10 training epochs. The Mosaic data augmentation technique involves randomly cropping and resizing four images, which are then spliced together into a single image for use in training data.

#### 2.5.2. Backbone Module

The backbone network is used for extracting target features, and the YOLOv8 backbone consists of the CBS convolution module, C2f module, and Spatial Pyramid Pooling Fast (SPPF) module.

(A) CBS convolutional module

**Figure 7:**
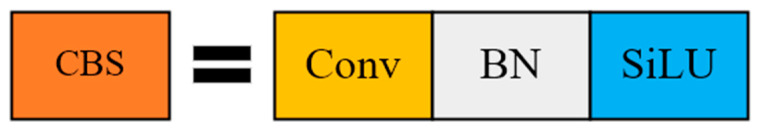
Convolution module.

The CBS convolution module consists of convolutional layers, batch normalization layers (BN), and the SiLU activation function, as illustrated in Figure 7.

The convolution operation involves traversing the image from the top left to the bottom right with a fixed stride, multiplying the pixel values covered by the fixed-size convolutional kernel at each position, and summing them up for computation. The result of this process is the feature map matrix. Common convolutional kernel sizes include 1 × 1 and 3 × 3, among others. The 3 × 3 convolution is used to expand the receptive field, while the 1 × 1 convolutional kernel is used to reduce the number of parameters. It is worth noting that the weight values of the convolutional kernel are shared across the entire image.

During model training, parameter updates can lead to changes in the output distribution of subsequent layers, thereby affecting the speed of model training. To address this issue, batch normalization techniques normalize the input data by mean and variance, ensuring a fixed distribution for each layer’s input data. This accelerates the gradient convergence speed of the model while preventing issues such as gradient vanishing and exploding.

The activation function used in the YOLOv8 model is the SiLU function, which is a special case of the Swish function [48] expressed as shown in Equation (1).

(1) $$Swish\left(x\right)=\frac{x}{1+exp(-\beta x)}$$

(B) C2f module

**Figure 8:**
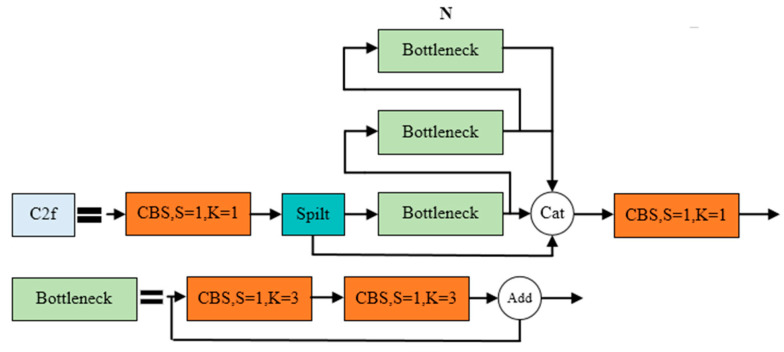
C2f module structure.

The C2f module is designed based on the C3 module of YOLOv5 and the concept of ELAN, controlling the gradient path length to achieve more effective learning and convergence. This allows the YOLOv8 model to obtain richer gradient information while maintaining its lightweight nature. The structure of the C2f module is illustrated in Figure 8.

(C) SPPF module

**Figure 9:**
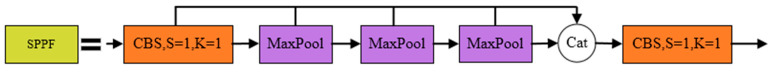
SPPF module.

The SPPF module transforms the parallel structure of the max-pooling layers into a serial structure and standardizes the convolutional layer size to 1 × 1. Experimental results demonstrate that this improvement further optimizes performance. The structure of the SPPF module is illustrated in Figure 9.

#### 2.5.3. Neck Module

The Neck section employs the Path Aggregation Feature Pyramid Network (PAFPN) [49] structure for multiscale feature fusion, aiming to integrate features from different levels to extract richer information.

#### 2.5.4. Head Module

**Figure 10:**
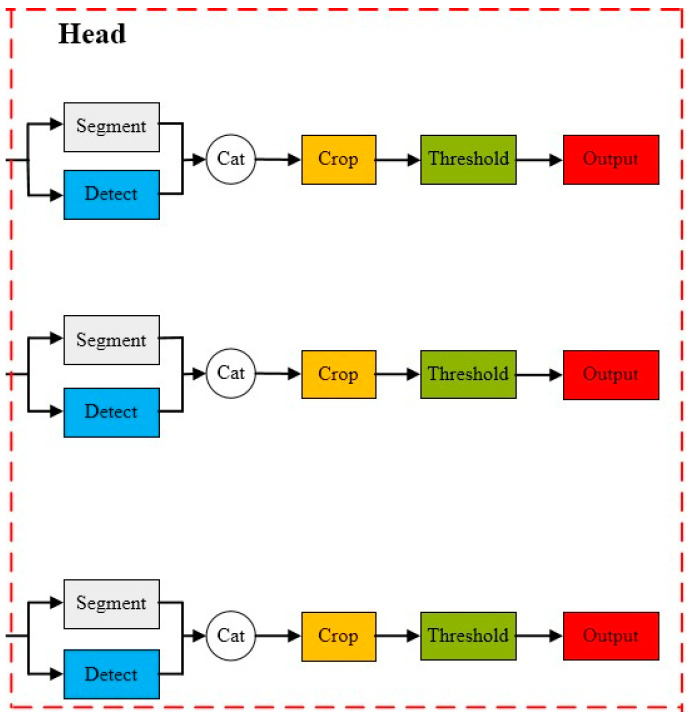
Detect Head.

YOLOv8 is a top–down instance segmentation model based on object detection, where the Head section decomposes the instance segmentation task into two parallel branch tasks. The image segmentation branch utilizes a fully convolutional network to generate a set of prototype masks, while the object detection branch performs bounding box prediction and class prediction. Additionally, each predicted box is encoded with a set of mask coefficients to represent instances. Subsequently, the two branches are combined through linear combination calculation to output instance masks, generating prototype masks as illustrated in Figure 10.

### 2.6. Evaluation Indicators

This study aims to achieve the localization and segmentation of each young maize plant with the ultimate goal of addressing practical challenges. Consequently, this study utilized the mean average precision (mAP) as the accuracy evaluation metric. The mAP50 (IoU of 50) and mAP50-95 (average precision at IoU ranging from 0.5 to 0.95 with a step size of 0.05), served as primary indicators for assessing model accuracy.

The precision rate (P) denotes the probability that a predicted positive example is actually a positive sample, as shown in Equation (2).

(2) $$P=\frac{TP}{TP+FP}$$

where TP is the number of correctly detected maize samples and FP is the number of incorrectly detected maize samples.

Recall (R) is a metric of model detection generalization, expressed as the proportion of correctly detected labels to the total true labels, as shown in Equation (3).

(3) $$R=\frac{TP}{TP+FN}$$

where FN is the number of undetected maize samples.

Mean average precision (mAP) represents the average of the network’s AP values for all categories in the dataset. The specific calculation expression is shown in Equation (4).

(4) $$mAP=\frac{1}{m}\sum AP(i)$$

where m is the number of labeled categories, AP is the area under the P-R curve, which responds to how well the model recognizes a category, and the P-R curve is the curve drawn based on the P and R values of the category.

### 2.7. Experimental Environment

The experimental platform for this study employs the Windows 10 operating system, an Intel (R) Core (TM) i9-9900K CPU processor with a main frequency of 3.60 GHz, and a GeForce RTX 2080 Ti GPU with 11 GB of video memory. The model training framework used is Pytorch 2.0.1, with specific training parameters detailed in Table 3:

## 3. Experiments and Analysis of Results

### 3.1. Comparison and Analysis of Different Instance Segmentation Models

To verify the effectiveness of the YOLOv8 model, three distinct band combinations were selected for experimentation: RGB (Red, Green, Blue), NER (Near-Infrared, Red Edge, Red), and NRG (Near Infrared, Red, Green). In the experiments, we compared six instance segmentation network models including YOLOv8, Mask R-CNN [50], Mask Scoring R-CNN [51], PointRend [52], and YOLOv5 [53] models. The experimental results are shown in Table 4, and there are some differences in different band combinations in different algorithmic models. Overall, the NRG and NER bands exhibit similar accuracy across all models. However, in the Cascade Mask R-CNN model, the NRG band outperforms the NER band significantly. Particularly in the YOLOv8 model, the NRG band achieves the highest accuracy among all bands and across all models. In contrast, the RGB band’s performance was generally lower than that of the other two bands across all models. This discrepancy may arise from the varying sensitivities of the bands to feature characteristics coupled with the disparate capabilities of algorithmic models to process band information.

In this experimental study, the YOLOv8 model exhibits excellent accuracy, which is attributed to its advanced deep learning one-shot detection framework. This framework processes images rapidly, accurately identifying young maize seedling plants. The YOLOv5 model also uses the one-shot detection framework, but the YOLOv8 model uses a C2f structure with a richer gradient flow and adjusts the number of channels differently for different scales of the model. The Head section of the YOLOv8 model transitioned from a coupled-head to a decoupled head structure, thereby segregating the classification and detection heads, and shifting from Anchor-Based to Anchor-Free. These changes may be the reason why the YOLOv5 model is ranked second. The PointRend model achieves more accurate segmentation through pixel-level rendering technology, enabling the model to process the image at a fine-grained level and improve segmentation accuracy. In contrast, the Mask R-CNN and Mask Scoring R-CNN detection frameworks rely on anchors and result in higher computational complexity. This may affect their segmentation accuracy when dealing with complex scenes. The Cascade Mask R-CNN model, a multi-stage detection model, has its final outcome influenced by the performance at each stage. Although this model may exhibit high accuracy in some cases, it is computationally expensive and may be less applicable in resource-limited environments. Furthermore, the multi-stage detection process means the final outcome is contingent upon each stage’s performance, potentially explaining lower accuracy levels.

In summary, both the band combination and model design greatly influence the performance of instance segmentation in remote sensing images. Future research could focus on optimizing the model structure to enhance both the accuracy and efficiency of instance segmentation.

### 3.2. Analysis of Overcrowded Seedling Plants

**Figure 11:**
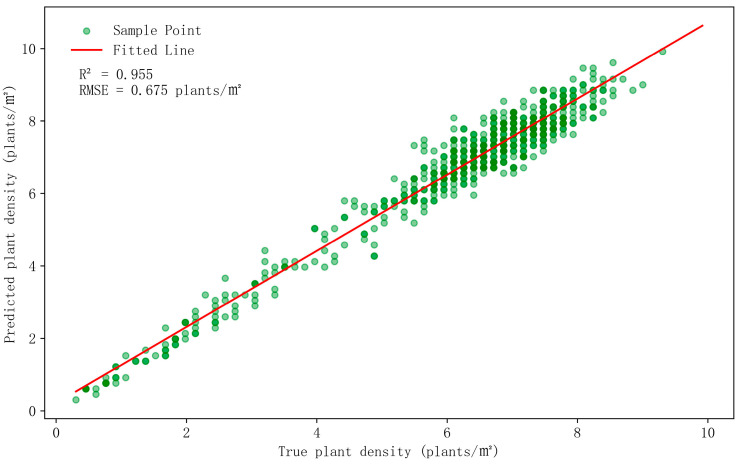
Comparison of model-detected plant density with real plant density.

During maize cultivation, planting density, an important agronomic parameter, directly affects yield and quality [54,55]. In this study, we employed the YOLOv8m model to assess the planting density of maize in Study Area B and analyzed the relationship between the detected planting density and the actual planting density, as illustrated in Figure 11. The variation in color depth within the figure represents the superposition effect of planting density; darker areas suggest that multiple locations share the same planting density, leading to an overlap of points and deeper colors. The results demonstrate that the YOLOv8m model exhibits high detection accuracy and reliability.

Based on the model’s strong performance, we further used the YOLOv8m model for the study of overcrowded zones. Overcrowding may lead to various issues, including heightened competition for light, nutrients, and water among plants, an increased risk of pests and diseases, and reduced efficiency of field operations. Thus, the identification and analysis of overcrowded zones in maize is imperative. The NRG-band image, which performs better in the YOLOv8 model, was chosen for the experiment to achieve the precise instance segmentation of young maize seedling plants, aiming to obtain the precise location information of each maize plant. By calculating the coordinates of the center point of each maize plant and taking into account the spacing criteria recommended by the maize field management program, we formulated a corresponding numerical specification to determine the circle’s size. Per the field management program, maize plants should be spaced between 25 and 30 cm apart. Given the spatial resolution of the remote sensing data utilized, we calculated the circle’s radius to be 62.5 pixel units, serving as the foundation for mapping the circular area centered on each maize plant’s center point. Additionally, we evaluated the probability of other plants’ presence or absence within each circular area to gauge planting density. To identify regions of high planting density, we set a probability threshold above which areas were considered densely planted. To visually represent areas of high planting density, we utilized cross-filament markers to denote them. In the future, this technology is anticipated to be deployed on drones or other monitoring devices for the real-time monitoring of maize plantations.

**Figure 12:**
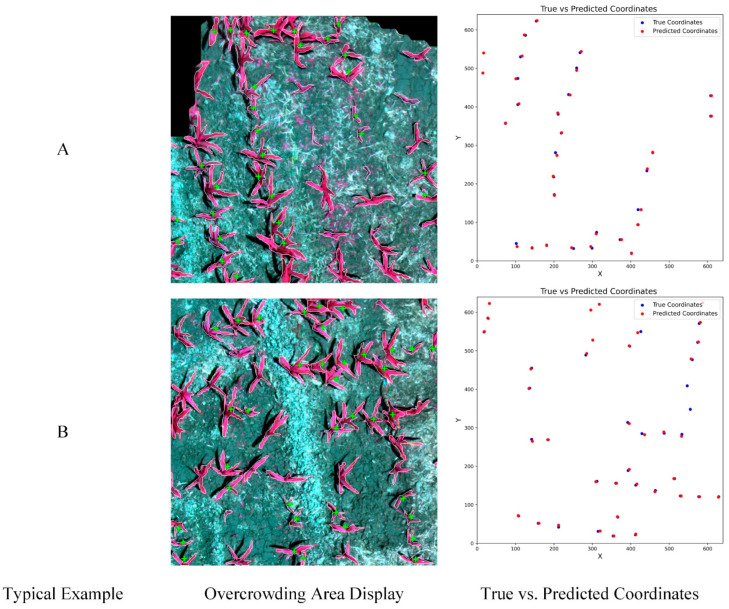
Overcrowding area display. (**A**) Image edge area; (**B**) Internal area of image. The green + is the overdense area detected by the model.

The specific effect is illustrated in Figure 12. The image depicts the result of the YOLOv8m model recognition using NRG remote sensing image, and it illustrates the positional relationship between the real coordinates and the predicted coordinates. The white outline lines represent individual maize seedling plants identified by the model, while the areas indicated by the cross-filaments highlight overdense areas identified by the model. These zones were automatically defined by the model based on the density of the seedling plants, emphasizing areas where field management might be required to optimize plant growth conditions.

By carefully comparing the recognition results, we can observe that some recognition results have some deviations from the real situation, and these deviations are mainly reflected in the recognition accuracy of the model for maize seedling plants. Sometimes, the model failed to accurately recognize independent maize seedling plants but misjudged the seedling plants with closer growth as a single plant, which led to the misreporting of overcrowded areas. This phenomenon may be caused by the model’s algorithm’s lack of accuracy in handling subtle densification differences between plants.

Despite these biases, the YOLOv8 model has demonstrated overall utility in recognizing the density of maize seedling plants. However, to enhance recognition accuracy, future research should consider developing a more comprehensive database and refining the model to better adapt to complex field conditions. This approach will enable agricultural workers to make more informed field management decisions, optimize crop growth conditions, and enhance yields.

## 4. Discussion

This section outlines three experiments conducted to explore the factors influencing the segmentation accuracy of maize instances. First, we examined the effect of varying parameter counts in the YOLOv8 model on segmentation accuracy. Second, experiments were conducted to assess the generalization capability of various mainstream instance segmentation models in Study Area B. Finally, we explored the performance of different spatial resolutions in the YOLOv8 model. The results and analysis of these experiments provide critical insights and guidance for advancing maize instance segmentation technology.

### 4.1. Effect of Different Parametric Quantities on Segmentation Accuracy

Five model configurations (n, s, m, l, and x) and three band combination methods (NRG, NER, and RGB) of YOLOv8 underwent training and validation. The results are presented in Table 5, where the input image sizes were consistently 640 × 640. This illustrates that regardless of the band combination, the mean average precision (mAP) demonstrates an increasing trend with the model’s complexity. However, with the model’s increasing complexity, there is also an increase in detection time, the number of floating point operations (GFLOPs), and the parameter count. Using the NRG band as an example, in comparison to the YOLOv8n model, the subsequent four models (s, m, l, and x) exhibited improvements of 2.8%, 4.4%, 5.7%, and 6.1% in object detection and 2.8%, 4.2%, 4.6%, and 4.8% in semantic segmentation, respectively. Notably, the YOLOv8m and YOLOv8x models perform similarly in detection and segmentation with a significant difference in FLOPs at 234.1 G and in parameter size at 44.512 MB. Given the YOLOv8m model’s strong performance in inference time, detection accuracy, and parameter count, it is recommended as a foundational model for ongoing research applications.

Second, the table reveals that the accuracy of the RGB band is notably lower than that of the NER and NRG bands. For example, with the YOLOv8m model, the mAP^Box^_50-95_ and mAP^Seg^_50-95_ of the NRG band reached 0.794 and 0.618, respectively. In contrast, although the inference speed increased by 0.8 ms, the detection and segmentation accuracy of the NER band saw a minor decline. Although few studies have directly elucidated the superiority of NRG bands across all relevant domains, existing research and remote sensing theories offer indirect support for this perspective. Given the high sensitivity of NRG bands to vegetation [56,57,58,59,60,61] and soil [62,63,64], their broad application in various areas [65,66,67,68,69,70,71], and utility in calculating vegetation indices [72,73,74,75,76,77,78,79,80], selecting NRG bands is a reasonable and effective strategy for routine studies in maize fields. Future studies will employ NRG bands as the primary bands to deliver reliable solutions for agricultural applications.

### 4.2. Generalization Experiment

The dataset constructed in Study Area B was utilized for generalization experiments. Six mainstream instance segmentation models, including YOLOv8, were chosen for the experiments, and the results are depicted in Table 6. These results unequivocally indicate that the YOLOv8 model presents considerable advantages over other algorithms. Although YOLOv8 possesses a greater number of parameters and higher computational requirements compared to the YOLOv5 model, its detection and segmentation accuracies (50-95) saw improvements of 3.1% and 3.4%, respectively, for the same model size (M model). This emphasizes the superiority of YOLOv8 in terms of overall performance, stability, and reliability, demonstrating its greater adaptability and generalizability. The YOLOv8 model is likely to offer greater advantages in various complex scenarios and tasks.

In addition, the inference time per image is decreased by 30.3 milliseconds relative to the PointRend model, despite the higher FLOPs of the YOLOv8m model. Detection accuracy and segmentation accuracy (50–95) have seen improvements of 15.7% and 11.4%, respectively. These results thoroughly validate the YOLOv8m model’s excellent performance in detection and segmentation tasks.

**Figure 13:**
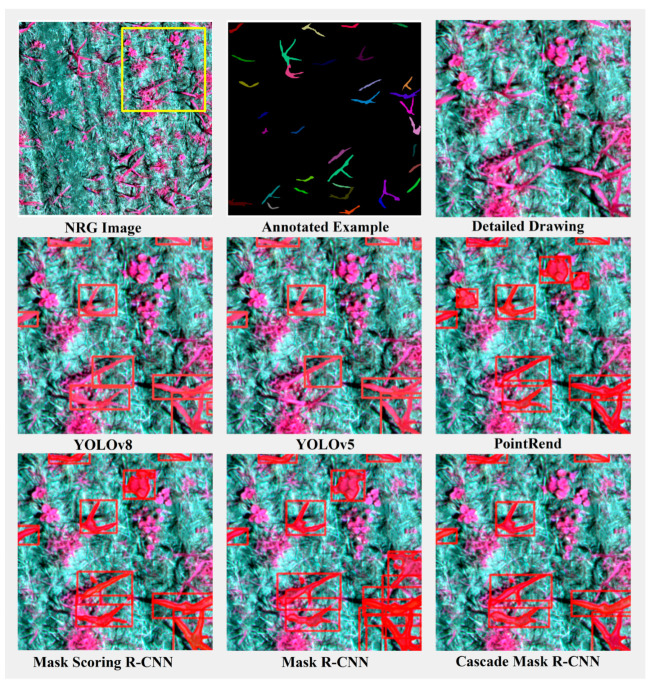
Comparison of segmentation results of model instances in the scenario of short, sparse and mixed weeds in maize. The red box represents the object detection box. The yellow box represents the area of detail enlargement.

**Figure 14:**
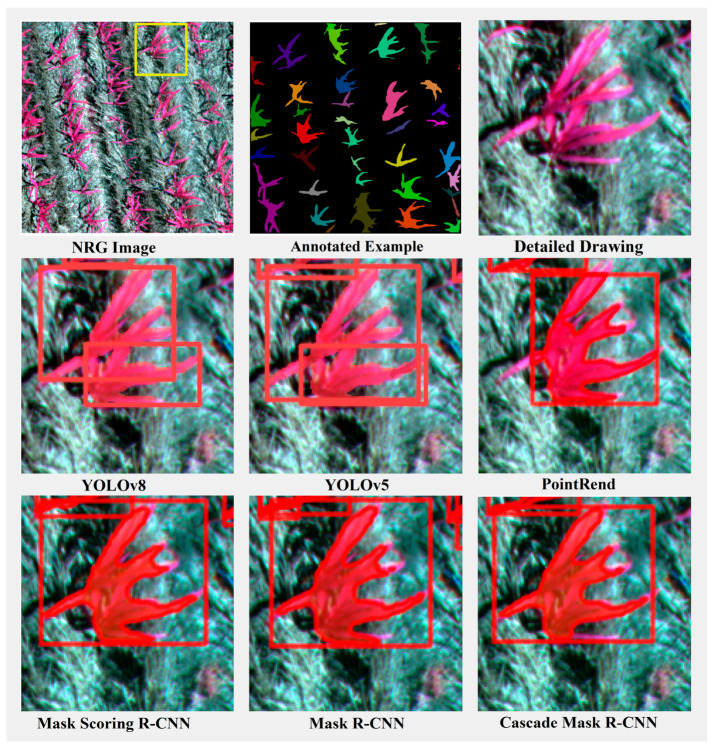
Comparison of segmentation results of model instances under dense maize growth scenario. The red box represents the object detection box. The yellow box represents the area of detail enlargement.

**Figure 15:**
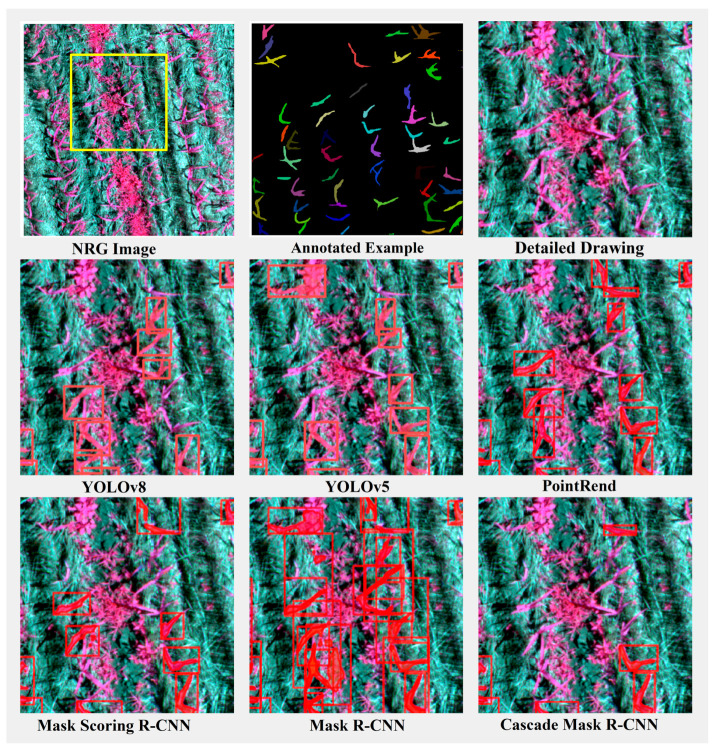
Comparison of segmentation results of model instances in scenarios with normal maize growth and mixed with other plants. The red box represents the object detection box. The yellow box represents the area of detail enlargement.

To more effectively illustrate the performance of different instance segmentation methods and visualize the detection results of each model, we selected three representative scenarios from the dataset for illustration: a scenario with relatively short, sparse maize mixed with weeds (Figure 13); a scenario with densely growing maize (Figure 14); and a scenario with normally growing maize mixed with other plants (Figure 15). These three scenarios encompass various conditions encountered during maize growth and facilitate a comprehensive evaluation of the performance of different instance segmentation models.

In this scenario, where the maize is relatively short, sparse, and mixed with weeds, the challenge lies in the model’s ability to distinguish between maize plants and weeds while accurately segmenting each instance despite the sparse distribution of plants. As Figure 13 illustrated in the figure, the PointRend, Mask Scoring R-CNN, and Mask R-CNN models exhibit misdetection by erroneously identifying other plants in the upper right corner as maize. In contrast, the YOLOv8 model demonstrates superior performance in recognizing and segmenting thin and elongated leaves of maize. However, all models exhibit a degree of under-detection for maize plants adjacent to weeds, especially for those that are short in height.

In the case of dense maize growth, the leaves overlap heavily, and the model must be able to handle this complexity and segment each instance accurately. For this reason, we deliberately chose the case of dense maize plants for demonstration. As can be seen in Figure 14, with the exception of the YOLOv8 model, all other models have the situation of recognizing two maize plants as one and fail to segment the maize leaves accurately and finely.

In the case where the maize grows normally and is mixed with other plants, the model must be able to effectively discriminate between different types of plants and accurately segment them into instances. The results, depicted in Figure 15, reveal that even the YOLOv5 model exhibits mixed detection omissions in this scenario, while the Cascade Mask R-CNN model shows substantial areas of omission. Particularly in scenes featuring a significant mixture of other plants in the middle, all models exhibit some degree of missed detection. Nevertheless, as evident from the figure, YOLOv8 exhibits the lowest leakage rate and achieves fine segmentation of the recognized corn plants’ leaves.

The YOLOv8 model demonstrated robust performance and generalization capabilities despite the variation in maize varieties between Study Area A and Study Area B. The YOLOv8 model excelled in detecting and generalizing across maize varieties. Its superiority over other algorithms lies in its ability to effectively adapt to different maize varieties while maintaining high detection and segmentation accuracy. Consequently, it is concluded that the YOLOv8 model is exceptionally adaptable and robust, handling maize varieties with varying characteristics and offering a reliable solution for agricultural applications. It is worth noting that although the YOLOv8 model performed best in the experiments, there is still room for improvement in the accuracy of segmenting the boundaries of a single maize plant especially when dealing with elongated leaves and mixed weeds. This finding offers valuable insights for future research directions and innovations.

### 4.3. Model Performance at Different Spatial Resolutions

The initial data for this study were derived from aerial images acquired by a UAV flying at an altitude of 8.5 m with a ground spatial resolution (GSD) of 0.4 cm/pixel. A primary advantage of this altitude is its capability to capture high-resolution images, thus offering clearer and more detailed data to support the instance segmentation of young maize. These high-precision data facilitate a more accurate identification and segmentation of plant instars during data annotation, thereby ensuring the reliability and accuracy of the study results. However, we recognize that such low flight altitudes may pose practical challenges in managing large areas. To identify the optimal imaging altitude for segmenting young maize instars and enhance the detection performance in real-world applications, we adjusted the spatial resolution to explore the relationship between spatial resolution and instance segmentation accuracy. The YOLOv8m model served as the base model, with the NRG band—demonstrating superior performance in previous experiments—selected for further experimental analysis, as detailed in Table 7.

**Figure 16:**
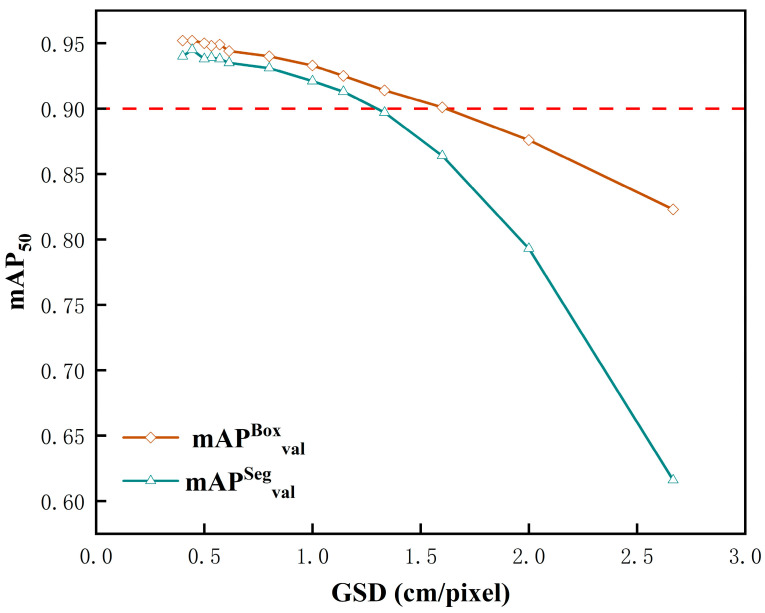
Instance segmentation accuracy at different spatial resolutions.

To more clearly illustrate the relationship between spatial resolution and instance segmentation accuracy, mAP50 is utilized as an indicator, and a line graph depicting the change in instance segmentation accuracy at various spatial resolutions is presented in Figure 16. The figure clearly shows that both detection accuracy and segmentation accuracy decrease with diminishing spatial resolution. As spatial resolution declines from 1.333 to 1.600 cm/pixel, there is a significant drop in accuracy—the mAP^Box^_50_ decreased by 1.3% and the mAP^Seg^_50_ decreased by 3.3%.

At a GSD of 1.333 cm/pixel, mAP^Box^_50_ achieves 91.4%, and mAP^Seg^_50_ achieves 89.7%, fulfilling the accuracy requirements for routine applications. Per Equation (5), with a GSD of 1.333 cm/pixel, the corresponding height (H) is 28.326 m, meeting the demands of large-scale daily scenarios for monitoring and managing young maize growth.

(5) $$H=\frac{f\times GSD}{a}$$

## 5. Conclusions

To achieve the precise positioning and segmentation of young maize seedlings in daily large-area scenarios, facilitating the monitoring and management of maize growth conditions, this study focuses on employing the YOLOv8 model for maize instance segmentation in UAV-based remote sensing. We systematically explore the impact of different multispectral band combinations, spatial resolutions, and model parameters on segmentation accuracy. The main conclusions are as follows:

(1) Combined with the SAM image segmentation large-scale model, it can realize the efficient annotation of young maize seedling plants. This approach facilitates the creation of a representative maize seedling plant instance segmentation dataset, addressing current research challenges like annotation data inaccuracy and training data limitations.

(2) The YOLOv8 model shows excellent segmentation accuracy under different multispectral data combinations and different resolutions, especially in NRG synthetic data; the accuracy of mAP^Box^_50-90_ reaches 0.794 and that of mAP^Seg^_50-95_ reaches 0.618. In the generalization experiments, YOLOv8 also performs well, which proves its applicability and stability under different environments and conditions. 

(3) Spatial resolution has a significant effect on the segmentation accuracy of young maize instances at a given flight altitude. Appropriate resolution can meet the needs of practical applications, which in turn improves the cost-effectiveness of applications in real-world scenarios. This finding is of great practical importance for monitoring and managing the growth of young maize in everyday large area scenarios.

This study offers a crucial theoretical foundation and practical guidance for deploying UAV remote sensing technology in agricultural automation and intelligent monitoring. In particular, the excellent performance of the YOLOv8 model in the generalization experiment further proves its great potential in the field of precision agriculture. Subsequent research will be devoted to optimizing the network structure to improve the segmentation detection ability of the model by adding the attention mechanism and changing the loss function. Meanwhile, we will also explore methods such as model pruning and knowledge distillation to reduce the number of parameters in order to improve the usability of the model in the case of limited computational power. Through these methods, we expect to further improve the accuracy and efficiency of segmentation of young maize seedling plant instances and provide effective technical support for precision agriculture.

## Figures and Tables

**Table 1:** Time cost of manual and SAM-based annotations.

Method	Average Time to Annotate on Particle (s)
Manual annotation	28.43
SAM-based semi-automatic annotation	5.5

**Table 2:** Statistics on large, medium and small instances.

Dataset	Class	Instances	Target Amount		
			Small	Medium	Large
Train Set	Maize	19,310	1620	931	16,759
Val Set	Maize	4754	389	197	4168
Test Set	Maize	29,226	2278	1360	25,588

**Table 3:** Model training parameters.

Parameter Name	Parameter Value	Parameter Name	Parameter Value
Epochs	200	Batch Size	2
Momentum	0.937	Weight_Decay	0.0005

**Table 4:** Comparison of segmentation accuracies of different band combination data and different models.

Model	Band	Box		Seg		Parameters (MB)
		mAP^val^_50_	mAP^val^_50-95_	mAP^val^_50_	mAP^val^_50-95_	
YOLOv8m	NRG	0.952	0.794	0.94	0.618	27.240
	NER	0.951	0.793	0.94	0.615	
	RGB	0.949	0.769	0.932	0.567	
YOLOv5m	NRG	0.952	0.788	0.942	0.611	26.531
	NER	0.952	0.786	0.941	0.609	
	RGB	0.947	0.755	0.933	0.56	
PointRend	NRG	0.918	0.665	0.897	0.524	55.755
	NER	0.918	0.666	0.906	0.524	
	RGB	0.909	0.631	0.895	0.463	
Mask Scoring R-CNN	NRG	0.906	0.639	0.891	0.499	60.230
	NER	0.913	0.638	0.892	0.498	
	RGB	0.904	0.6	0.88	0.435	
Mask R-CNN	NRG	0.910	0.637	0.895	0.511	43.971
	NER	0.915	0.640	0.902	0.515	
	RGB	0.903	0.596	0.884	0.454	
Cascade Mask R-CNN	NRG	0.805	0.619	0.782	0.437	77.021
	NER	0.777	0.605	0.764	0.429	
	RGB	0.766	0.576	0.379	0.752	

**Table 5:** YOLOv8 model (n, s, m, l, x) in different bands’ instance segmentation results.

Model	Band	Box		Seg		Speed (ms)	Parameters (MB)	FLOPs (G)
		mAP^val^_50_	mAP^val^_50–95_	mAP^val^_50_	mAP^val^_50–95_			
YOLOv8n	NRG	0.942	0.75	0.928	0.576	11.7	3.263	12.1
	NER	0.941	0.751	0.928	0.579	12.4		
	RGB	0.937	0.718	0.919	0.524	14.1		
YOLOv8s	NRG	0.947	0.778	0.937	0.604	20.9	11.790	42.7
	NER	0.949	0.777	0.935	0.601	23.4		
	RGB	0.946	0.744	0.931	0.551	22.3		
YOLOv8m	NRG	0.952	0.794	0.94	0.618	30.8	27.240	110.4
	NER	0.951	0.793	0.94	0.615	30.0		
	RGB	0.949	0.769	0.932	0.567	31.1		
YOLOv8l	NRG	0.954	0.807	0.941	0.623	36.2	45.937	220.8
	NER	0.953	0.806	0.943	0.622	36.1		
	RGB	0.95	0.778	0.936	0.575	38.6		
YOLOv8x	NRG	0.952	0.811	0.942	0.624	59.9	71.752	344.5
	NER	0.953	0.812	0.943	0.626	59.6		
	RGB	0.952	0.79	0.94	0.584	58.8		

**Table 6:** Comparison of accuracy of different instance segmentation models on test set (NRG images).

Model	Box		Seg		Speed (ms)	FLOPs (G)	Parameters (MB)
	mAP^test^_50_	mAP^test^_50-95_	mAP^test^_50_	mAP^test^_50-95_			
YOLOv8m	**0.795**	**0.585**	**0.788**	**0.462**	25.2	110.4	27.240
YOLOv5m	0.772	0.554	0.763	0.428	26.1	95.4	26.531
PointRend	0.73	0.428	0.714	0.348	55.5	90.299	55.755
Mask Scoring R-CNN	0.725	0.411	0.698	0.327	47.6	183.296	60.230
Mask R-CNN	0.701	0.389	0.682	0.322	66.7	145.408	43.971
Cascade Mask R-CNN	0.602	0.387	0.582	0.285	43.5	240.64	77.021

**Table 7:** Example segmentation accuracy at different spatial resolutions.

Model	Band	GSD (cm/Pixel)	Box		Seg	
			mAP^val^_50_	mAP^val^_50–95_	mAP^val^_50_	mAP^val^_50–95_
YOLOv8m	NRG	0.400	0.952	0.794	0.94	0.618
		0.444	0.952	0.794	0.945	0.62
		0.500	0.95	0.784	0.938	0.61
		0.533	0.948	0.783	0.939	0.605
		0.571	0.949	0.778	0.938	0.604
		0.615	0.944	0.773	0.935	0.598
		0.800	0.94	0.75	0.931	0.573
		1.000	0.933	0.722	0.921	0.548
		1.143	0.925	0.696	0.913	0.526
		1.333	0.914	0.668	0.897	0.494
		1.600	0.901	0.623	0.864	0.442
		2.000	0.876	0.562	0.793	0.356
		2.667	0.823	0.464	0.616	0.232

## Data Availability

The data presented in this study are available on request from the corresponding author. The data are not publicly available due to privacy.
